# Medication Management of Early Pregnancy Loss in an Urban Texas Emergency Department

**DOI:** 10.1111/acem.70293

**Published:** 2026-04-27

**Authors:** Lindsay Cline, Emilie Sandfeld, Haley Brunkal, Madeline Fischer, Allison Gilbert, Lauren Fine, Emily E. Ager

**Affiliations:** ^1^ Texas A&M University College of Medicine Bryan Texas USA; ^2^ Independent consultant Philadelphia PA; ^3^ Department of Obstetrics and Gynecology Baylor University Medical Center Dallas Texas USA; ^4^ Department of Emergency Medicine Baylor University Medical Center Dallas Texas USA; ^5^ Department of Emergency Medicine University of California San Francisco San Francisco California USA; ^6^ National Clinician Scholars Program University of California San Francisco San Francisco California USA

**Keywords:** early pregnancy loss, miscarriage, reproductive health, spontaneous abortion

## Abstract

**Background:**

Among patients with early pregnancy loss (EPL) without medication complications or need for urgent surgical evacuation, treatment should largely be guided by patient preference. However, evidence describing medication management for EPL in the emergency department (ED) setting is limited. We examine the occurrence of medication management of EPL with misoprostol in an urban Texas ED, describe associated clinical outcomes, and identify patient and clinical factors associated with offering this treatment.

**Methods:**

This was a retrospective study of patients with confirmed EPL at a single urban academic ED in Texas from November 1, 2022, to May 31, 2024. Data were collected via relevant EHR review. Eligible patients were identified via ICD‐10 codes and reviewed using a structured abstraction tool. We identified patients who received expectant or medication management with misoprostol and reported clinical outcomes using descriptive statistics. An exploratory multivariable logistic regression was used to identify characteristics predictive of patients being offered misoprostol.

**Results:**

During the study period, 181 patients met our inclusion criteria. Most patients (*n* = 154; 85.1%) received expectant management. Misoprostol was offered to 44 patients (24%); 27 patients (15.0%) received the medication, all of whom had an OB consult. Seven‐day return ED visits were low in both the expectant (*n* = 18; 11.5%) and medication (*n* = 2; 7.4%) management groups. Of the 18 patients managed expectantly with a 7‐day return ED visit, six received misoprostol during the second visit. OB consultation strongly predicted patients being offered misoprostol (aOR 15.1; 95% CI 4.8–47.61).

**Conclusion:**

Medication management was rarely provided to ED patients with confirmed EPL; OB was consulted for all patients who received misoprostol. Return ED visits were rare among patients managed expectantly and with misoprostol. Several patients received misoprostol during a return ED visit, which may suggest a missed opportunity for medication treatment of EPL during initial ED presentation.

## 1 Introduction

### 1.1 Background

Early pregnancy loss (EPL), defined as a nonviable, intrauterine pregnancy within the first 12 6/7 weeks of gestation, is common and occurs in approximately 10%–20% of all recognized pregnancies [1]. Management options for EPL include expectant management, medication management with misoprostol with or without mifepristone, or procedural management with uterine aspiration. All approaches are safe and effective among clinically stable patients.

Given the importance of patient autonomy in navigating a pregnancy loss, a patient‐centered approach is crucial and treatment choice should be guided largely by patient preference [1, 2, 3]. Factors such as cost, duration of symptoms, invasiveness, and location of care may influence patient preferences. For some patients, medication or procedural management may be preferred over expectant management due to the shorter symptom duration, decreased time to miscarriage completion, and potentially fewer follow‐up visits [4, 5, 6, 7, 8, 9]. Medications may be preferred over uterine aspiration by some patients given the lower cost and noninvasiveness [10].

For medication management of EPL, pretreatment with mifepristone, when available, followed by misoprostol is recommended [1]. However, mifepristone is not available in all healthcare settings due to restrictive state and federal policies, along with institutional factors including formulary restrictions and pharmacy practice inertia, even in states without significant legal barriers [11, 12]. In these contexts, a misoprostol‐only regimen may be the most feasible approach [13, 14]. Compared to expectant management, misoprostol alone results in accelerated uterine evacuation and a reduced need for surgical intervention [5, 6, 7, 9]. While expectant management results in spontaneous completion by 14 days in approximately 70% of cases, misoprostol alone increases completion to 84% by 8 days [4, 5]. This effect is more pronounced in specific EPL subtypes: for anembryonic gestations, completion rates improve from 25% to 81% and for missed abortion (“asymptomatic EPL”) from 30% to 88% [4, 5].

### 1.2 Importance

Emergency department (ED) utilization for EPL is common, with an estimated 900,000 annual encounters for suspected or confirmed EPL [15]. Despite some potential advantages over expectant management, medication management of EPL is uncommon in the ED setting [7, 8, 9]. Recent evidence suggests only 5.4% of commercially insured ED patients with confirmed EPL received medication management compared to 11.2% of patients seen in ambulatory settings [7]. Though patient preference may play a role, clinician knowledge, time restrictions, and lack of EPL‐specific protocols are likely contributing factors [16].

With the increasing prevalence of US counties with no or limited obstetric services—so‐called “maternity care deserts”—ED utilization for early pregnancy‐related care may increase. Half of Texas counties are currently classified as maternity care deserts [17, 18, 19]. Further, patients seeking EPL‐related care in the ED are more likely to be publicly insured, Black, or Hispanic compared to reproductive age women seeking ED care for other reasons [15, 20, 21]. Restrictive abortion policies disproportionately impact these patients and further decrease access to comprehensive reproductive health care [22].

The ED represents an important setting for EPL care, particularly for underserved and marginalized communities [2]. However, no studies describe the characteristics and clinical outcomes of patients who receive medication management versus expectant management for confirmed EPL in US EDs. It remains unclear what factors guide emergency medicine and consulting obstetric clinicians in deciding whether to offer medication management. In addition to potential clinical benefits, such as a shorter time to EPL resolution and decreased need for procedural intervention, ensuring all eligible patients are offered active management can support patient autonomy and inform a patient‐centered approach to EPL care.

### 1.3 Goals of This Investigation

The objective of this study is to examine the occurrence of medication management for EPL in the ED and describe associated clinical outcomes. We Also explore patient and visit characteristics predictive of being offered medication management compared to expectant management for EPL.

## 2 Methods

### 2.1 Study Design and Setting

This was a retrospective study of ED patients with a confirmed EPL diagnosis at a single urban, academic level 1 trauma center in Texas From November 1, 2022, to May 31, 2024. The study site ED sees approximately 110,000 patients per year. In this ED there is no standardized requirement for an OB consult in order to provide misoprostol, and interdepartmental guidance supports emergency physician‐led medication management for many patients with EPL. Data was obtained via manual review of the electronic health record (EHR). The study was approved by the Baylor Scott & White Research Institute Institutional Review Board. This study adheres to STROBE guidelines for observational studies [23].

### 2.2 Participant Selection and Data Collection

We identified potentially eligible patients using a broad set of relevant *International Classification of Diseases*, *10th Revision* diagnosis codes informed by prior literature on this topic and the authors' clinical expertise: O03 (spontaneous abortion), O02 (missed abortion), O04 (complications following induced termination), and O07 (failed attempted termination) [15, 20, 21]. Manual chart abstraction to identify patients with confirmed EPL eligible for medication management was then performed independently by three trained research assistants, all of whom received training on the use of the electronic data collection instrument (Supporting Information Appendix 1). Determination of diagnoses included review of clinical data, including serum quantitative β‐hCG and ultrasound results, as well as narrative documentation by the ED care team, including the history of present illness, physical exam, and medication decision making. If there was uncertainty regarding an EPL diagnosis, a second trained chart abstractor reviewed the EHR. To address the anticipated diagnostic uncertainty related to “pregnancies of unknown location,” all encounters given this diagnosis by the research assistant were then jointly reviewed by an attending emergency physician (L.F.) and an attending OB/GYN (A.B.) until diagnostic agreement was reached. The principal investigator monitored all data collection activities and provided a final review if there were disagreements or discrepancies between chart abstractors.

We excluded patients not eligible for medication management of EPL, including those with prior use of misoprostol or mifepristone documented at the index ED encounter, abortive or procedural intervention prior to or at the index ED visit, or a bleeding disorder (including hemophilia, Von Willebrand disease, or Factor V Leiden), which is a contraindication to receiving misoprostol [1]. Patients requiring operative intervention at the index visit were excluded as the need for urgent surgical management precluded consideration of medication management. We also excluded patients with ectopic pregnancy, pregnancy of unknown location, complete abortion, and pregnancy loss at a gestation of 13 weeks or greater.

After identifying patients with confirmed EPL using standardized definitions and diagnostic criteria, we categorized cases as incomplete abortion, missed abortion, or anembryonic pregnancy (Table 1) [24, 25]. When available, radiology‐performed ultrasound results were reviewed to determine EPL type.

**TABLE 1:** Diagnostic definitions: Included versus excluded early pregnancy loss diagnoses.

Included/excluded	Diagnosis	Definition
Included	Incomplete abortion	Ultrasound or other evidence of retained pregnancy tissue, visual inspection of fetal tissue, or high clinical suspicion of incomplete pregnancy loss based on history and physical exam. No cardiac activity or other evidence of viability.
	Missed abortion	Fetal or embryonic demise with gestational tissue in the uterus. Typically, little or no vaginal bleeding and evidence that the nonviable gestation has remained in the uterus for a period of time.
	Anembryonic pregnancy	Gestational sac > 25 mm without embryo or yolk sac.
Excluded	Threatened abortion	Vaginal bleeding with evidence of embryonic or fetal viability, such as fetal cardiac activity.
	Pregnancy loss > 12 weeks 6 days	Any pregnancy loss where products, or LMP if products were unavailable, were dated ≥ 13 weeks.
	Pregnancy of unknown location	Positive pregnancy test with transvaginal ultrasound that shows neither an IUP nor an ectopic pregnancy.
	Ectopic pregnancy	A pregnancy implanted in an abnormal location.
	Complete abortion	History of early pregnancy loss with an empty uterus.
	Unable to categorize	Information within patient's chart is insufficient for confirming a diagnosis.

Abbreviations: EPL, early pregnancy loss; IUP, intrauterine pregnancy; LMP, last menstrual period.

Gestational duration was estimated using ultrasound‐determined crown‐rump length (CRL) or gestational sac (GS) measurements. If both measurements were available, the CRL was utilized. If ultrasound imaging was unavailable or unable to determine gestational duration, clinician documentation of patient‐reported LMP was used. If LMP was unknown, documentation of a patient‐reported gestational duration was used. When dating discrepancies existed between LMP and ultrasound dating, ultrasound measurements were utilized. Further details on ultrasound determination of diagnosis are included in Supporting Information Appendix 1.

The index visit of an EPL episode was defined as the first ED encounter during which EPL was diagnosed. The majority of index visits occurred at the study site; patients whose index visit for EPL was at an outside facility (with return ED visits to the study site) were considered a potential study subject if their outside hospital records were accessible through our EHR. If a patient had multiple EPL episodes during the study period, only the first episode was included in our analysis to avoid statistical bias from non‐independent events.

### 2.3 Outcomes

We collected patient demographics, visit characteristics, and clinical outcomes during ED encounters for confirmed EPL. Patient information included age, race, ethnicity, insurance payer, gravity, and parity. In the study site EHR, race and ethnicity are self‐reported. For the index EPL ED visit, we recorded a patient's EPL diagnosis type (incomplete abortion, missed abortion, or anembryonic pregnancy), gestational duration, presence of an OB consult, documentation of clinicians offering misoprostol, and receipt of misoprostol. Clinical outcomes included receipt of misoprostol, receipt of a blood transfusion, need for operative intervention, and hospitalization.

We also identified EPL‐related return ED visits within seven and 30 days, which were chosen as reasonable timelines for patients to experience immediate or delayed EPL‐related symptoms or complications necessitating evaluation. A return visit was considered related to the index ED visit if the encounter concerned EPL‐related care, as determined by the following EHR elements: clinician documentation, gestational duration, and documented gravity and parity. We also report clinical management during repeat ED visits, including receipt of β‐hCG testing, misoprostol, procedural intervention, blood transfusion, and hospitalization.

### 2.4 Primary Data Analysis

Our analysis was conducted at the individual patient level. We report descriptive statistics of the study population, with continuous variables presented as medians with interquartile range (IQR) and categorical data presented as frequencies and proportions. We used descriptive statistics to summarize misoprostol administration, dosing, and route of administration, as well as the occurrence of return ED visits, subsequent EPL‐related treatment, blood transfusion, and hospitalization. As an exploratory analysis, we used multivariable logistic regression analysis to examine patient and encounter characteristics predictive of patients being offered medication management, including age, race, ethnicity, insurance coverage, gestational duration, and OB consultation. Misoprostol being offered rather than administered was chosen given the strong component of patient preference in the receipt of this treatment. We report unadjusted and adjusted estimates. All analyses were conducted using Stata 19 (StataCorp, College Station, TX). We report odds ratios (OR) and associated 95% confidence intervals.

## 3 Results

### 3.1 Study Population Demographic and Clinical Characteristics

**FIGURE 1:**
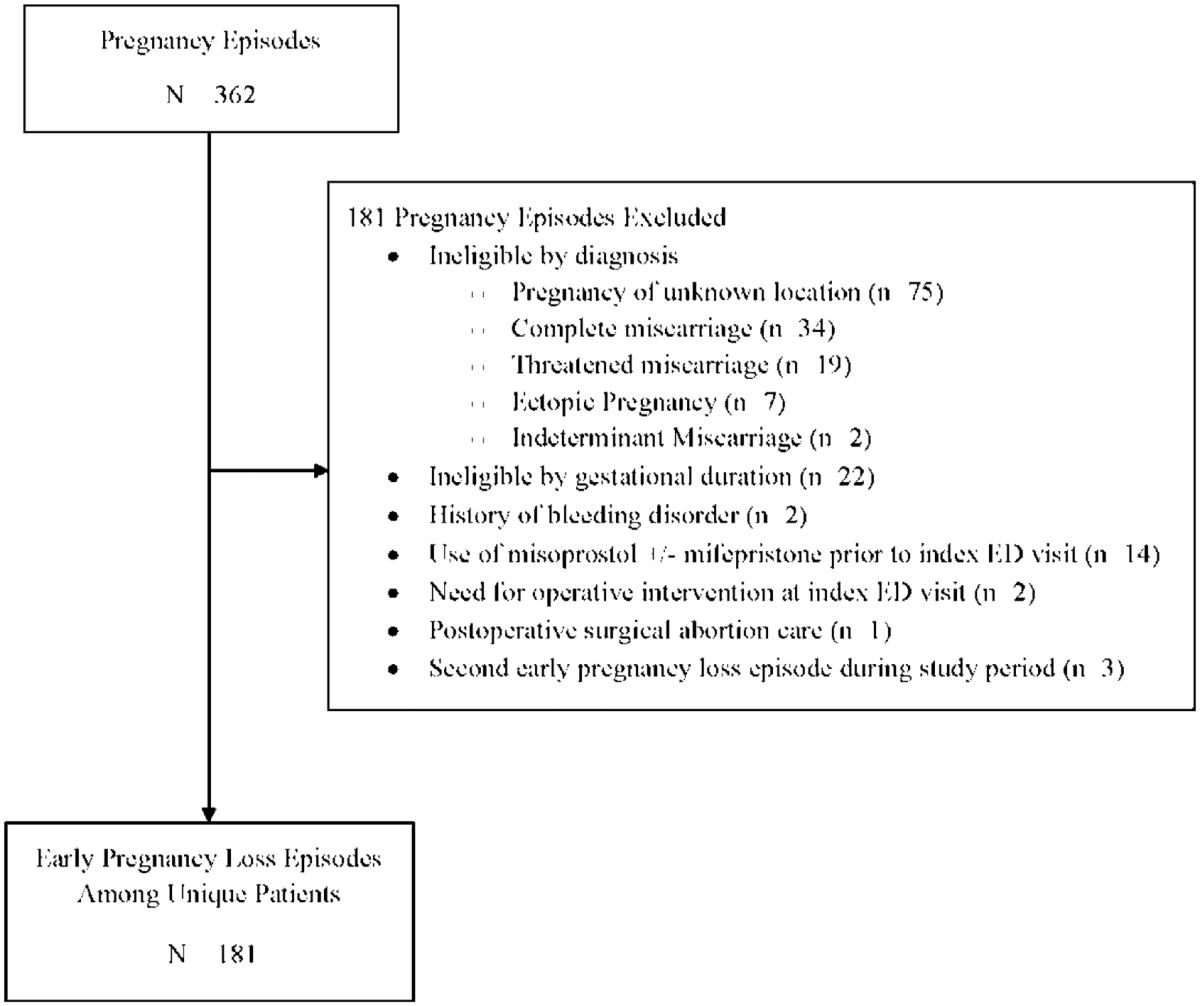
Determination of study population.

During the 19‐month study period, 362 pregnancy episodes among 355 patients were electronically identified for screening. After accounting for exclusion criteria, 181 unique patients were included in the analysis. Participant selection and reasons for exclusion are described in detail in Figure 1.

The median age was 28 among both patients who received misoprostol (IQR 24–34) and the expectant management (IQR 23–32) group; 146 patients (80.7%) were less than 35 years old. Overall, 93 (51.4%) patients were White, 71 (39.2%) were Black or African American, 9 (5.0%) were another race, and 8 (4.4%) had an unknown race. Seventy‐nine patients (43.7%) were Hispanic. Seventy‐four patients (40.9%) had commercial insurance, 70 (38.7%) were publicly insured (Medicaid or Tricare), and 37 (20.4%) were uninsured or self‐paid. Patient demographics by EPL management type are presented in Table 2.

**TABLE 2:** Demographics and clinical characteristics of emergency department patients with early pregnancy loss.

Characteristic	Patients, no. (%)		
	Total, *N* = 181	Misoprostol group, *n* = 27 (14.9%)	No misoprostol group, *n* = 154 (85.1%)
Age (years), median (IQR)	28 (23–32)	28 (24–34)	28 (23–32)
Age (years)			
< 35	146 (80.7)	21 (77.8)	125 (81.2)
≥ 35	35 (19.3)	6 (22.2)	29 (18.8)
Race			
Black/African American	71 (39.2)	9 (33.3)	62 (40.3)
White	93 (51.4)	16 (59.3)	77 (50.0)
Other	9 (5.0)	1 (3.7)	8 (5.2)
Unknown	8 (4.4)	1 (3.7)	7 (4.6)
Ethnicity			
Hispanic	79 (43.7)	10 (37.0)	69 (44.8)
Not Hispanic	97 (53.6)	16 (59.3)	81 (52.6)
Unknown	5 (2.8)	1 (3.7)	4 (2.6)
Insurance coverage			
Private	74 (40.9)	13 (48.2)	61 (39.6)
Public (Medicaid or Tricare)	70 (38.7)	7 (25.9)	63 (40.9)
Self‐pay or uninsured	37 (20.4)	7 (25.9)	30 (19.5)
Gravity, median (IQR)	3 (2–4)	2 (2–4)	3 (2–4)
Parity, median (IQR)	1 (0–2)	1 (0–2)	1 (0–2)
Diagnosis			
Incomplete abortion	166 (91.7)	27 (100)	139 (90.3)
Missed abortion	13 (7.2)	0	13 (8.4)
Anembryonic pregnancy	2 (1.1)	0	2 (1.3)
Gestational duration			
< 9w0d weeks	101 (55.8)	17 (63.0)	84 (54.6)
9w0d–12w6d	80 (44.2)	10 (37.0)	70 (45.5)
Ultrasound status			
Performed, products datable	57 (31.49)	8 (29.63)	49 (31.82)
Performed, products not datable	121 (66.85)	19 (70.37)	102 (66.23)
No ultrasound performed	3 (1.66)	0	3 (1.95)
Gestational duration determination			
Ultrasound	57 (31.5)	8 (29.6)	49 (31.8)
Last menstrual period	124 (68.5)	19 (70.4)	105 (68.2)

Abbreviation: IQR, interquartile range.

Most patients were diagnosed with incomplete EPL (*n* = 166; 91.7%), followed by missed EPL (*n* = 13; 7.2%), and anembryonic pregnancy (*n* = 2; 1.1%) (Table 2). All patients in the misoprostol group were determined to have an incomplete EPL. In the expectant management group, 139 patients (90.3%) were diagnosed with an incomplete EPL, 13 (8.4%) had a missed EPL, and two (1.3%) were diagnosed with an anembryonic pregnancy. Overall, 178 patients (98.3%) received ultrasound imaging: 57 had datable products of conception and 121 had undatable products of conception. Gestational duration was therefore determined by ultrasound for 57 patients (31.5%) and by LMP for 124 patients (68.5%). Most patients (*n* = 101; 55.8%) had a gestational duration less than nine weeks. See Table 2.

### 3.2 Main Results

Of the 184 included patients, 44 (24.3%) had clinician documentation of offering misoprostol to patients: 43 patients with an incomplete abortion (25.9%), one patient with a missed abortion (7.7%), and no patients with an anembryonic pregnancy. Twenty‐seven patients (14.9%) received misoprostol and 154 (85.1%) were managed expectantly. Overall, 100 patients (54.3%) had a documented OB consult. Of the patients with an OB consult, 40 (40.0%) were offered misoprostol, compared to four patients (4.9%) without an OB consult. All 27 patients who received misoprostol had an OB consult, compared to 73 patients (47.4%) managed expectantly. See Table 3.

**TABLE 3:** Clinical management and outcomes of emergency department patients with early pregnancy loss.

Characteristic	Patients, no. (%)		
	Total, *N* = 181	Misoprostol group, *n* = 27 (14.9%)	No misoprostol group, *n* = 154 (85.1%)
Offered misoprostol			
Yes	44 (24.3)	27 (100)	17 (11.0)
No	137 (75.7)	0	137 (89.0)
Obstetrics consult			
Yes	100 (54.3)	27 (100)	73 (47.4)
No	81 (45.8)	0	81 (52.6)
Index visit clinical management			
Blood transfusion	1 (0.6)	0	1 (0.7)
Admitted	1 (0.6)	0	1 (0.7)
EPL‐related return ED visit, ≤ 7 days	19 (10.5)	2 (7.4)	17 (11.0)
Misoprostol administration	6 (3.3)	0	6 (3.9)
Only hCG	4 (2.2)	0	4 (2.6)
Blood transfusion	1 (0.6)	0	1 (0.6)
Surgical intervention (OR)	1 (0.6)	0	1 (0.6)
Hospital admission	0	0	0
EPL‐related return ED visit, 8–30 days	6 (3.3)	2 (7.4)	4 (2.6)
Misoprostol administration	0	0	0
Only hCG	0	0	0
Blood transfusion	0	0	0
Surgical intervention (OR)	1 (0.)	0	1 (0.6)
Hospital admission	0	0	0

Abbreviations: ED, emergency department; EPL, early pregnancy loss; OR, operating room.

Among the medication management group, 25 patients (92.6%) were offered misoprostol by the OB consult physician. Seventeen patients (63.0%) received misoprostol in the ED, three (11.1%) received misoprostol in the ED and were prescribed an additional dose if needed, and seven (25.9%) were prescribed the medication to take at home. Misoprostol dosing varied, with 16 patients (59.3%) receiving 800 mcg, four (14.8%) receiving 600 mcg, and seven (25.9%) receiving 400 mcg. The route of administration also varied: 17 (63.0%) oral, 7 (25.9%) vaginal, 2 (7.4%) buccal, and 1 (3.7%) sublingual. See Table 4.

**TABLE 4:** Encounter and clinical characteristics of emergency department patients with early pregnancy loss who received medication management.

	*N* = 27, no. (%)
Offering clinician	
Emergency medicine	1 (3.7)
OB/GYN	25 (92.6)
Unknown	1 (3.7)
Ordering clinician	
Emergency medicine	11 (40.7)
OB/GYN	15 (55.6)
Unknown	1 (3.7)
Location of administration	
ED only	17 (63.0)
Prescription only	7 (25.9)
ED and prescription	3 (11.1)
Misoprostol dose	
400 mcg	7 (25.9)
600 mcg	4 (14.8)
800 mcg	16 (59.3)
Misoprostol administration	
Oral	17 (63.0)
Vaginal	7 (25.9)
Buccal	2 (7.4)
Sublingual	1 (3.7)

Among the entire study population, 19 patients (10.5%) had a return ED visit within seven days. This included 17 patients (11.0%) in the expectant management group and two patients (7.4%) in the medication management group (Table 3). Among the 17 patients managed expectantly who had a 7‐day return visit, six (35.3%) received misoprostol during their second visit; none of these patients were offered misoprostol at their index ED visit. No patients in the misoprostol group received misoprostol during the second ED visit. No patients in either group received a blood transfusion, surgical intervention, or were required to have hospital admission during a subsequent ED visit. See Table 4.

Six patients had a return ED visit between 8 and 30 days after the index ED visit: two (7.4%) in the misoprostol group and four (2.6%) in the expectant management group. One patient in the expectant management group received procedural intervention during this visit; no patients received misoprostol, required a blood transfusion, or were admitted.

Results of our exploratory multivariable logistic regression are presented in Table 5. Patient demographics, including age, race, ethnicity, and insurance payer, did not predict if a patient was offered medication management during the index ED visits, nor did gestational duration. An OB consult was associated with a patient being offered misoprostol, with an unadjusted OR of 12.8 (95% CI 4.35, 37.86) and adjusted OR of 15.1 (95% CI 4.78, 47.61).

**TABLE 5:** Results of exploratory multivariable logistic regression: Predictors and characteristics of emergency department patients being offered medication management with misoprostol for early pregnancy loss.

	Unadjusted		Adjusted	
	Odds ratio	95% Confidence interval	Odds ratio	95% Confidence interval
Age				
< 35 years old	Reference		Reference	
≥ 35 years old	1.10	(0.47, 2.56)	0.96	(0.35, 2.62)
Race				
White	Reference	—	Reference	—
Black/African American	0.47	(0.22, 1.01)	0.32	(0.09, 1.14)
Other	0.66	(0.13, 3.39)	0.33	(0.05, 2.24)
Ethnicity				
Not Hispanic	Reference	—	Reference	—
Hispanic	1.16	(0.58, 2.32)	0.46	(0.14, 1.52)
Insurance				
Private	Reference	—	Reference	—
Self‐pay or uninsured	1.07	(0.45, 2.54)	1.93	(0.65, 5.76)
Public (Medicaid or Tricare)	0.52	(0.23, 1.16)	0.82	(0.31, 1.28)
Gestational duration				
< 9w0d weeks	Reference	—	Reference	—
9w0d–12w6d	0.65	(0.32, 1.31)	0.57	(0.25, 1.28)
OB consult				
No	Reference	—	Reference	—
Yes	12.83	(4.35, 37.86)	15.08	(4.78, 47.61)

## 4 Discussion

To our knowledge, this is the first US ED‐based study using all‐payer data to examine the occurrence of medication management for EPL and associated clinical outcomes. In this single‐center, retrospective study of ED patients with EPL, only 15% received medication management. Furthermore, less than one‐quarter of patients, all of whom were eligible for medication treatment, had documentation in the EHR indicating that misoprostol was offered.

This low utilization may reflect several factors, including clinician knowledge and comfort with medication management of EPL, perceptions of treatment urgency, and expected clinical benefit. Although most patients (*n* = 166; 92%) were diagnosed with incomplete abortion, only 43 (26%) were offered misoprostol. Notably, among patients with missed abortion (*n* = 13; 7%)—those most likely to benefit from medication management—only one patient was offered misoprostol, and none received the medication. While professional guidelines support shared decision‐making and the use of misoprostol as an evidence‐based option for EPL, our findings and prior literature suggest that this strategy remains underutilized in ED practice [1, 26]. Though not explored in this study, expanding the use of medication management may help improve autonomy and patient satisfaction for patients seeking EPL care in the ED [27, 28].

Among patients who received misoprostol, the dosing and route varied. ACOG recommends an initial dose of 800 micrograms of vaginal misoprostol for medication management of EPL; however, multiple misoprostol dosing regimens and routes of administration have been described in the literature [1]. The observed variation in this cohort likely reflects this evidence‐supported heterogeneity rather than deviation from clinical guidelines [7, 29, 30]. Lastly, ACOG acknowledges that a combined regimen of mifepristone and misoprostol, compared to misoprostol alone, is preferred for managing EPL [1]. Mifepristone was not available at the study site ED during the study period. Institutions should continue to advocate for access to this medication, as robust evidence and national clinical guidelines support the use of the combined regimen [1, 13, 14, 31].

Return ED visits within 7 days were low in both the expectant and medication management groups. Among the 17 patients who were initially managed expectantly and had a 7‐day return visit, approximately one‐third received misoprostol during the second ED visit. None of these patients had evidence of being offered misoprostol at their index visit. This finding suggests a possible missed opportunity to offer medication treatment to patients with a pregnancy loss. In a randomized trial of immediate versus delayed medication management for EPL in a French gynecologic ED, delayed treatment was associated with a near doubling of unplanned ED visits compared to immediate medication treatment with mifepristone and misoprostol (34.1% vs. 16.9%) [32]. Prolonged pregnancy loss symptoms can cause an undue physical and emotional burden on patients for a condition that already carries a high occurrence of adverse mental health outcomes [33]. Such unplanned visits can also increase overall EPL‐related healthcare expenditures.

Importantly, reasons for returning to the ED may not be related to emergent complications, though all return visits in our study population were related to the index EPL encounter. Qualitative studies find that patients often feel under‐informed about their EPL diagnosis in the ED setting, resulting in confusion, anxiety, and unplanned return visits [27, 28, 34]. Patients may also seek ED care when access to other settings is limited, such as on weekends and holidays. Lastly, nearly two‐thirds of our study population were publicly insured, uninsured, or self‐pay. Financial and access barriers may lead patients to seek follow‐up care in the ED, regardless of their initial treatment.

A higher proportion of patients with an OB consult were offered misoprostol compared to those without a consult (40% vs. 5%, respectively). Additionally, results from our exploratory multivariable regression demonstrated that documentation of an OB consultation strongly predicted that a patient would be offered misoprostol. While we accounted for major patient demographic and clinical characteristics, residual confounders may have influenced this finding. As there is no departmental requirement for OB involvement for medication management of EPL, OB consultation may reflect unfamiliarly and/or discomfort with providing medication this care among emergency clinicians. However, our observed complication rates, including blood transfusion, uterine aspiration, or hospitalization, were low and consistent with existing literature [6, 9, 26, 29, 30, 31]. This finding suggests medication management is safe and effective for emergency clinicians to provide. For example, a 2014 randomized controlled trial comparing misoprostol to expectant management found that hemorrhage requiring transfusion occurred in 1.1% of both groups, emergent surgical intervention occurred in 2.1% vs. 2.2% of patients, and hospitalization in 3.2% vs. 4.4% of patients, respectively [6]. Our findings may help reassure emergency clinicians who are hesitant to use misoprostol due to unfamiliarity or concerns regarding complications.

## 5 Limitations

This study has several important limitations. First, this was a retrospective, single‐site study with a small sample size. Although our EHR query identified 362 encounters, a substantial number were excluded during eligibility screening, leaving 181 patients, only 44 of which were offered misoprostol. This small sample size in the medication management group limited our ability to identify patient and clinical factors associated with clinicians offering patients misoprostol.

Additionally, ascertainment of our outcomes of interest was limited to documentation available within our EHR. Our institution uses Epic Systems, and additional encounter data were accessible through the Care Everywhere health information exchange. However, encounters occurring at facilities outside this network were not captured, potentially leading to underestimation of outcomes in the entire study population. However, the majority of EDs in the metro area of the study site utilize Epic Systems and would be visible through Care Everywhere. Additionally, no direct patient follow‐up was conducted, which limits the ability to assess patient‐centered outcomes such as patient experience, satisfaction, or time to symptom resolution, all of which are important patient‐oriented outcomes in EPL care.

Finally, medication management shortens the time to complete expulsion among patients with missed EPL or anembryonic pregnancy; however, these subgroups were underrepresented in our sample (7% and 1%, respectively), which limits our ability to assess treatment effects in these populations [4, 10, 13]. Among patients diagnosed with missed EPL or anembryonic pregnancy in this study, only one was offered misoprostol, suggesting a gap in care for patients who may benefit the most from medication management.

These limitations collectively constrain the generalizability of our findings, particularly to EDs with different workflows, OB consultation capabilities, patient populations, or prescribing practices. Nevertheless, the study contributes to evidence supporting the safety of ED‐based medication treatment of EPL and highlights the need for prospective, adequately powered research.

## 6 Conclusion

In this single‐site, ED‐based study, medication management of EPL was uncommon, with variable misoprostol dosage and route of administration. Less than a quarter of patients were offered medication treatment, and Obstetrics consultation significantly increased the odds of misoprostol being offered. Among patients managed with medications, complications and repeat ED visits were uncommon, supporting the safety of this treatment. Given our retrospective data and small sample size, future studies are needed to examine the impact of ED‐based medication EPL management on clinical outcomes, healthcare utilization, and patient satisfaction.

## Author Contributions

Study concept and design: Lauren Fine and Emilie Sandfeld. Data acquisition: Lauren Fine, Lindsay Cline, Emilie Sandfeld, and Haley Brunkal. Data analysis and interpretation: Emily E. Ager, Lauren Fine, and Madeline Fischer. Statistical analysis: Emily E. Ager. Manuscript drafting: Emily E. Ager, Lauren Fine, Lindsay Cline, and Haley Brunkal. Critical revision of manuscript: Emily E. Ager, Lauren Fine, and Allison Gilbert.

## Funding

This study was supported by the University of California San Francisco National Clinician Scholars Program (E.E.A.).

## Conflicts of Interest

The authors declare no conflicts of interest.

## Supporting information


**Data S1:** acem70293‐sup‐0001‐Supinfo01.docx.

## Data Availability

The data that support the findings of this study are available on request from the corresponding author. The data are not publicly available due to privacy or ethical restrictions.
