# Supplementary material for: Medication Management of Early Pregnancy Loss in an Urban Texas Emergency Department
Source: Acad Emerg Med. 2026 Apr 27;33(4):e70293. doi: 10.1111/acem.70293 (PMC13116052; doi:10.1111/acem.70293)
Supplement: Supplementary file 1 — Data S1: acem70293‐sup‐0001‐Supinfo01.docx. [file ACEM-33-0-s001.docx]

**Medication Management of Early Pregnancy Loss in an Urban Texas Emergency Department**

**Appendix 1.** Determination of Early Pregnancy Loss Diagnosis and Procedure for Assessing Inclusion/Exclusion Criteria:

1. Obtain MRN number from “MRN Master List-use to work from”
2. Login to Epic; Type in “DSH BUMC ED” as Department
3. Once in ED track board, select “ED Chart” in upper header (May need to first select “More” in Upper Header) and enter MRN Number
4. Select Patient and appropriate visit based on chief complaint related to miscarriage, vaginal bleeding in pregnancy, etc.
5. If several visits are present for one instance of potential miscarriage, follow the below protocol and determine if any of those visits could have allowed for misoprostol use based on diagnosis
6. Making the Diagnosis
   1. Select “Chart Review”, then select “Imaging” for the appropriate visit. Review “Findings” and” Impression” to assist in diagnosis classification using Chart A (below) and the provided definition set below.
   2. Review the HPI, physical exam findings, b-hCG value and medical management decision making from the ED provider note as needed to assist in classifying the pregnancy loss.
   3. If unsure of your diagnosis, enter “need” into the 2^nd^ reviewer slot for additional review by medical student. If diagnoses conflict after review or if either medical student is unable to reach a conclusive diagnosis, type “need” in the reviewer 3 column to request Dr. Fine’s review.
7. Confirm First Trimester
   1. Gestational age should be preferentially determined by ultrasound dating, if available. Select “Chart Review”, then “Imaging” from the appropriate visit and find gestational sac size or crown-rump length. If both are available, use dating from crown-rump length. If the products were not dated or undateable, the patient’s reported LMP will be used.
   2. Select “Chart Review”, then “Notes” and look for “ED Provider Note” from the appropriate visit. Within the HPI, look for the patient’s LMP to determine gestational age. If the LMP is not listed in the ED Provider note, check for a triage note or an OB-GYN consult note from the same visit.
   3. Enter the patient’s LMP in [https://reference.medscape.com/calculator/240/due-date-by-lmp](https://urldefense.com/v3/__https://reference.medscape.com/calculator/240/due-date-by-lmp__;!!KwNVnqRv!BQy27jXSxbcN8LCxJJADHguLr1KeURkBpiLRf3YH01-fshzoUIIYjrcUofrCNl8p5DYRZH_bb5jim91Vd5aW86E5$) to determine if the patient meets 1^st^ trimester criteria (12/6 or less).
8. Confirm No History of Bleeding Disorder
   1. Finally, to assess for history of bleeding disorder, go to “Snapshot” and check the patient’s “Problem List” on the right-hand side. If none (Von Willebrand, Hemophilia A/B, Factor V Leiden Mutation) are listed, then go to “Chart Review”, “Notes” and look for “ED Provider Note” from the index visit. Check within the HPI and within the Past Medical History section of the note to confirm there is no known history of bleeding disorder.
9. Screening Diagnosis Definitions with Corresponding Number Code
   1. **Pregnancy loss >12 weeks 6 days (1)** - Any pregnancy loss where products, or LMP if products were unavailable, were dated ≥ 13 weeks.
   2. **Missed Miscarriage (2) -** Fetal or embryonic demise with gestational tissue in the uterus. Typically, little or no vaginal bleeding and evidence that the nonviable gestation has remained in the uterus for a period of time.
   3. **Incomplete EPL (3) -** Ultrasound or other evidence of retained pregnancy tissue with history of or ongoing vaginal bleeding. No cardiac activity or other evidence of viability. (3)
   4. **Pregnancy of Unknown Location (PUL) (5) -** Positive pregnancy test with transvaginal ultrasound that shows neither an IUP nor an ectopic pregnancy.
   5. **Threatened Miscarriage (6) -** Vaginal bleeding with evidence of embryonic or fetal viability, such as fetal cardiac activity. Has not progressed to a state from which recovery is impossible (potentially reversible); see Chart A for specific criteria
   6. **Complete Miscarriage (7) –** History of early pregnancy loss with an empty uterus.
   7. **Indeterminate Miscarriage (8) -** Information available on patient’s charts is insufficient for confirming a diagnosis.
   8. **Anembryonic pregnancy (9) -** Gestational sac >25mm without embryo or yolk sac.
   9. **Ectopic Pregnancy (10) -** A pregnancy implant in an abnormal location.
10. Eligibility Column
    1. Eligible: If the diagnosis number is 2, 3, or 9, confirmed first trimester, confirmed that there is no history to bleeding disorder, and does not meet any additional exclusion criteria as listed below: Mark as eligible
    2. Ineligible: any diagnosis 1, 5, 6, 7, 8, 10
    3. Ineligible: Confirmed > 12 weeks and 6 days gestation
    4. Ineligible: Confirmed to have history of bleeding disorder
    5. Ineligible: Confirmed to have received operative management at index visit
    6. Ineligible: Confirmed to have received mifepristone, misoprostol, or abortive operative intervention for pregnancy of interest prior to index visit
11. If eligible, list the date for the index visit that corresponds with your diagnosis
12. Fill in excel doc labeled “Miso Data Collection-Work on this one” with your findings, decision on inclusion vs exclusion of the patient, and brief description of reasoning
13. Update “Miso Patient Identifier Master” with the MRN and corresponding alphabetical tag (Patient ID)

**Ultrasound considerations:**

Ultrasounds were reviewed to help determine a patient’s diagnosis. If there was evidence from the HPI or from a previous ED visit of an intrauterine pregnancy (IUP) and the patient was now showing an empty uterus on ultrasound, the patient was diagnosed with a complete miscarriage and excluded from the study. Ultrasounds suspicious for an ectopic pregnancy were excluded from the study. Ultrasounds demonstrating CRL > 7mm with no fetal heart tones and minimal to no vaginal bleeding were diagnosed as missed miscarriages. Ultrasounds concerning for retained products of conception (heterogenous or echogenic material in the uterus or cervix) were deemed to represent incomplete miscarriage when the history and exam were also consistent. If ultrasound imaging demonstrated a gestational sac greater than 25 millimeters without an embryo or yolk sac, an anembryonic pregnancy was diagnosed. For patients who presented with vaginal bleeding and had an ultrasound with a viable fetus or a fetus of uncertain viability, threatened abortion was diagnosed. These cases were excluded from inclusion in the study as a definitive diagnosis of EPL could not be made.

**Additional Considerations:**

Given the anticipated diagnostic uncertainty inherent to PUL and the possibility of misclassification, after the initial reviewer made a diagnosis of PUL, **all** of these charts were then jointly manually reviewed by an attending emergency physician (LF) and an attending OB/GYN (AB). This attending review included the same review process: review of the emergency department record, obstetrics consultation notes, any available outpatient documentation, and review of all transvaginal ultrasound images. This review continued until was 100% agreement on the diagnoses between LF and AB. Based on the diagnosis from this review, cases that were instead determined to have an EPL diagnosis were included in our analysis; those that were indeed PUL were excluded from our analysis.

**Chart A:** Transvaginal Ultrasonographic Diagnosis of Pregnancy Failure in a Woman with an Intrauterine Pregnancy of Uncertain Viability

| **Findings Diagnostic of Pregnancy Failure** | **Findings Suspicious for, but Not Diagnostic of, Pregnancy Failure*** |
| --- | --- |
| Crown–rump length of ≥7 mm and no heartbeat | Crown–rump length of <7 mm and no heartbeat |
| Mean sac diameter of ≥25 mm and no embryo | Mean sac diameter of 16–24 mm and no embryo |
| Absence of embryo with heartbeat ≥2 wk after a scan that showed a gestational sac without a yolk sac | Absence of embryo with heartbeat 7–13 days after a scan that showed a gestational sac without a yolk sac |
| Absence of embryo with heartbeat ≥11 days after a scan that showed a gestational sac with a yolk sac | Absence of embryo with heartbeat 7–10 days after a scan that showed a gestational sac with a yolk sac |
|  | Absence of embryo ≥6 wk after last menstrual period |
|  | Empty amnion (amnion seen adjacent to yolk sac, with no visible embryo) |
|  | Enlarged yolk sac (>7 mm) |
|  | Small gestational sac in relation to the size of the embryo (<5 mm difference between mean sac diameter and crown–rump length) |

**Note:** Chart adapted from “Doubilet PM, Benson CB, Bourne T, Blaivas M. Diagnostic Criteria for Nonviable Pregnancy Early in the First Trimester. Campion EW, ed. N Engl J Med. 2013;369(15):1443-1451. doi:10.1056/NEJMra1302417.”
